# Latency reversal agents modulate HIV antigen processing and presentation to CD8 T cells

**DOI:** 10.1371/journal.ppat.1008442

**Published:** 2020-03-20

**Authors:** Julie Boucau, Jishnu Das, Neelambari Joshi, Sylvie Le Gall

**Affiliations:** 1 Ragon Institute of MGH, MIT and Harvard, Massachusetts General Hospital and Harvard Medical School, Cambridge, Massachusetts, United States of America; 2 Center for Systems Immunology, Departments of Immunology and Computational & Systems Biology, University of Pittsburgh School of Medicine, Pittsburgh, Pennsylvania, United States of America; Vaccine Research Center, UNITED STATES

## Abstract

Latency reversal agents (LRA) variably induce HIV re-expression in CD4 T cells but reservoirs are not cleared. Whether HIV epitope presentation is similar between latency reversal and initial infection of CD4 T cells is unknown yet crucial to define immune responses able to detect HIV-infected CD4 T cells after latency reversal. HIV peptides displayed by MHC comes from the intracellular degradation of proteins by proteasomes and post-proteasomal peptidases but the impact of LRAs on antigen processing is not known. Here we show that HDAC inhibitors (HDCAi) reduced cytosolic proteolytic activities while PKC agonists (PKCa) increased them to a lesser extent than that induced by TCR activation. During the cytosolic degradation of long HIV peptides in LRA-treated CD4 T cells extracts, HDACi and PKCa modulated degradation patterns of peptides and altered the production of HIV epitopes in often opposite ways. Beyond known HIV epitopes, HDACi narrowed the coverage of HIV antigenic fragments by 8-11aa degradation peptides while PKCa broadened it. LRAs altered HIV infection kinetics and modulated CD8 T cell activation in an epitope- and time-dependent manner. Interestingly the efficiency of endogenous epitope processing and presentation to CD8 T cells was increased by PKCa Ingenol at early time points despite low levels of antigens. LRA-induced modulations of antigen processing should be considered and exploited to enhance and broaden HIV peptide presentation by CD4 T cells and to improve immune recognition after latency reversal. This property of LRAs, if confirmed with other antigens, might be exploited to improve immune detection of diseased cells beyond HIV.

## Author summary

Latently HIV-infected CD4 T cells persist and remain invisible to the immune system. Strategies to flush out HIV reservoirs propose to re-express HIV with latency reversal agents (LRAs), leading to CD4 T cell death or clearance by HIV-specific immune responses. LRAs tested so far variably induced HIV re-expression but did not eliminate reservoirs. The activation of HIV-specific immune responses is triggered by HIV peptides displayed by infected cells after HIV intracellular degradation. Whether HIV antigens are similarly degraded and displayed by CD4 T cells after latency reversal or during initial infection is unknown. We showed that LRAs altered the activities of the degradation machinery and changed the degradation patterns of HIV into peptides. LRA-treated HIV-infected CD4 T cells were variably recognized by immune cells in a time- and peptide-dependent manner. Some LRAs increased the efficiency of HIV peptide presentation despite low levels of HIV antigens inside CD4 T cells. The modulation of HIV peptide presentation by current or future LRAs should be accounted for and exploited to improve HIV peptide presentation and enhance immune detection of HIV-infected CD4 T cells after latency reversal.

## Introduction

Despite efficient antiretroviral treatments (ART) HIV latently persists in long-lived CD4 T cells [1]. One of the eradication strategies currently tested in clinical trials proposes to re-activate provirus expression with latency reversal agents (LRAs) to trigger HIV re-expression, leading to cell death or elimination by pre-existing HIV-specific immune responses [2,3]. While HIV RNA re-expression can be induced to variable extents by LRAs in CD4 T cells ex vivo or in vivo, the reservoir is never cleared [4,5]. This failure of LRAs may be explained by limited reactivation of HIV provirus [6,7] and/or of antigen expression, mutations in epitopes impairing immune recognition, ineffective magnitude or functionality of immune effectors [8–11], or intrinsic resistance of cells to elimination [12,13]. Whether steps leading to HIV MHC-peptide presentation to immune cells are similar during productive infection or latency reversal is not known but play a major role in assessing the capacity of pre-existing immune responses to recognize CD4 T cells after latency reversal and in defining additional vaccine-induced immune responses relevant to latency reversal.

LRAs tested in vitro or in vivo in clinical trials [14] include HDAC inhibitors (HDACi) such as Vorinostat [15], Romidepsin [16,17] or Panobinostat [18], PKC agonists (PKCa) such as Bryostatin or Ingenol, aldehyde dehydrogenase inhibitor Disulfiram [19,20]. The addition of immunomodulatory components such as TLR agonists [21–24], PD1 blockade [25], cytokines such as IL-7 or IL-15 [26–28], activation of the RIG-I pathway [29], or of non-canonical NF-κB signaling activator [30] may enhance HIV latency reversal in ex vivo or animal studies but have not yet been tested in clinical trials or have not been successful.

Peptides displayed by MHC-I to CD8 T or NK cells derive from the intracellular degradation of proteins by the antigen processing machinery, including proteasomes and post-proteasomal peptidases in the cytosol and endoplasmic reticulum (ER) where LRA-induced HIV antigens may be found [31]. The expression and hydrolytic activities of the antigen processing machineries are modulated by various stimuli such as cytokines [32], viral infection [33], TLR ligands [34–36], TCR-induced cellular activation [37], oxidative stress [38] or enzyme inhibitors [39–43]. We previously showed that TCR-mediated cellular activation enhanced peptidase activities, modulated protein degradation patterns into peptides and CTL recognition [37]. The metabolic environment for HIV protein expression and degradation differs drastically between productive infection in activated CD4 T cells [44,45] and latency reversal in differentiated resting CD4 T cells [46,47], but the consequences on the degradation machinery and epitope production are not known.

Here we showed that HDACi and PKCa used in clinical trials modulate cellular peptidase activities of primary CD4 T cells in opposite ways. They variably altered degradation patterns of HIV long peptides, the production of known HIV epitopes often in opposite ways. HDACi narrowed the coverage of HIV antigenic fragments by 8-11aa-long degradation peptides while PKCa broadened it. LRAs modulated the efficiency of endogenous processing and presentation of HIV epitopes by HIV-infected CD4 T cells and CTL responses in a time- and epitope-dependent manner. Interestingly PKCa Ingenol increased the efficiency of peptide presentation to CTL at early time points despite low levels of antigens inside cells. Altogether the data show that the impact of LRAs on antigen presentation should be accounted for and exploited to enhance immune recognition of CD4 T cells after latency reversal and for the design of vaccines against reservoirs. Rather than aiming for strong HIV reactivation, augmenting or broadening HIV peptide presentation after LRA treatment despite low antigens production would improve immune detection. Beyond latency reversal of HIV, HDACi or PKCa improving or broadening MHC peptide presentation at low levels of antigens may facilitate the immune detection of diseased cells.

## Results

### Different classes of LRA alter cytosolic peptidase activities

We assessed the effects of 5 LRAs on the antigen processing machinery of primary CD4 T cells. The selected LRAs with confirmed *in vitro* and partial *in vivo* efficacy on HIV reactivation included HDACi Panobinostat (Pano) and Romidepsin (Romi); Disulfiram (Disu); and PKCa: Bryostatin (Bryo) and Ingenol-3-angelate (Inge) while anti-CD3/CD28 TCR stimulation defined the maximum cellular activation.

**Fig 1:**
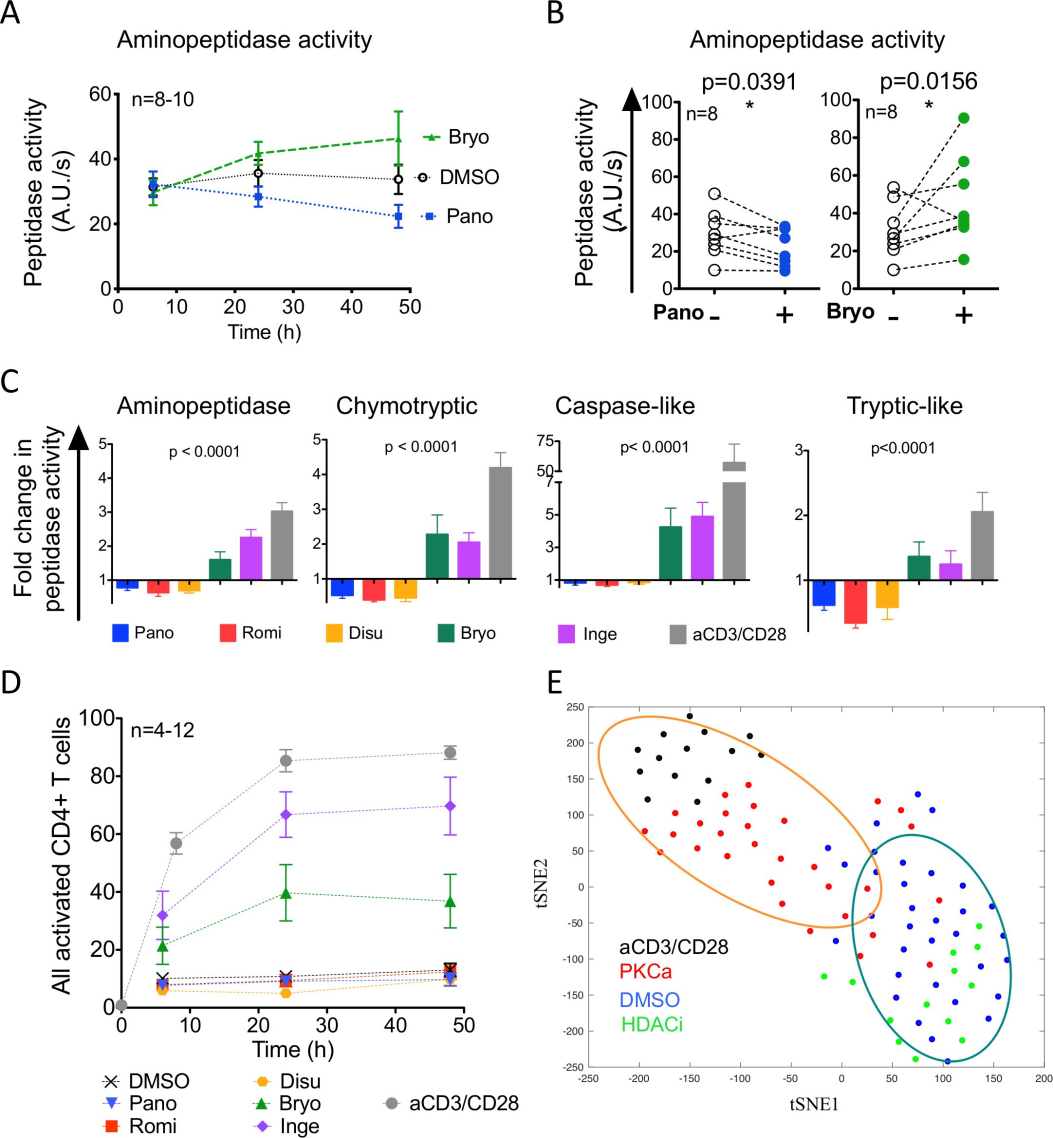
Different classes of LRA alter cytosolic peptidase activities. A. Aminopeptidase hydrolytic activity in primary CD4 T cells of healthy donors (n = 8–10) at 6, 24 and 48 h after treatment with DMSO (open circles), Bryostatin (green triangles) or Panobinostat (blue squares). B. Aminopeptidase hydrolytic activity in paired DMSO- (open circles) and Panobinostat-treated (blue circles, left) or DMSO- and Bryostatin-treated (green circles, right) primary CD4 T cells at 48 h post-treatment (n = 8 healthy donors). Wilcoxon matched-pairs signed-rank t tests analysis. C. Fold change in aminopeptidase, proteasomal chymotryptic, caspase-like, tryptic-like hydrolytic activities in LRA-treated over DMSO-treated cells at 48 hours: Panobinostat- (blue), Romidepsin- (red), Disulfiram- (orange), Bryostatin- (green), Ingenol- (purple) or aCD3/CD28-treated samples (n = 6–16). One-Way ANOVA Kruskal-Wallis tests on the 6 groups and p-values are reported. D. CD25 and CD69 surface levels were monitored by flow cytometry in primary CD4 T cells at 6, 24 and 48 h post treatment with DMSO (black crosses), Panobinostat (inverted blue triangles), Romidepsin (red squares), Disulfiram (orange circles), Bryostatin (green triangles), Ingenol (purple diamonds) or aCD3/CD28 (grey circles). n = 4–12 healthy donors. E. Unsupervised visualization with t-SNE on Z score centered and normalized data, which included the percentages of CD25-, CD69-, CD38 and HLA-DR-positive CD4 T cells in each sample and the values for aminopeptidase and proteasome chymotryptic, caspase-like and tryptic-like hydrolytic activities. Panobinostat and Romidepsin were grouped as HDACi (green, n = 13) and Bryostatin and Ingenol were grouped as PKCa (red, n = 30), also displayed are mock-treated DMSO (blue, n = 33) and aCD3/CD28-treated (black, n = 14). Ellipses indicate 75% confidence interval.

We measured with a fluorescence-based enzymatic assay the hydrolytic activities of the proteasome (chymotryptic, tryptic-like and caspase-like activities) and the post-proteasomal aminopeptidase activities involved in MHC-I presentation in live CD4 T cells of healthy donors at 6, 24 and 48 hours post-treatment [37] (Fig 1). Peptidase activities significantly changed after a 48 hours treatment (Fig 1A). Bryostatin treatment significantly increased aminopeptidase hydrolytic activities by an average 1.6-fold and Panobinostat significantly decreased hydrolytic activities by 0.78-fold (Fig 1A and 1B). Overall on 8–15 healthy donors, HDACi and disulfiram had opposite effects on peptidase activities of primary CD4 T cells when compared to PKCa or aCD3/CD28 (Fig 1C and S1 Fig). Treatment with HDACi or disulfiram significantly decreased the proteasomal chymotryptic-like, tryptic-like and caspase-like hydrolytic activities and aminopeptidase activities (0.35- and 0.85-fold depending on the activities). In contrast PKCa significantly increased aminopeptidase and proteasomal activities (2.05- to 4.9-fold increase depending on the activities) but to a lesser extent than TCR activation. Our previous work indicated that the increase in peptidase activities following aCD3/CD28-stimulation is positively correlated with cellular activation of primary CD4 T cells [37]. We assessed the expression of activation markers CD25 and CD69 at the surface of primary CD4 T cells following LRA-treatment (Fig 1D). Neither HDACi nor disulfiram changed the proportion of CD25^+^ and/or CD69^+^ primary CD4 T cells when compared to DMSO-treated cells but both PKCa increased the expression of the activation markers over time with a maximum of 40%-70% of activated CD4 T cells following PKCa treatments, remaining below the stimulation achieved with aCD3/CD28 (up to 88%), in accordance with previously reported PKCa-induced T cell activation measurements [48–50]. Unlike what we previously reported for cellular activation following CD3/CD28 engagement, the changes in peptidase activities following LRA treatment were not necessarily correlated with the percentage of activated cells. The multivariate analysis of all four peptidase activities and activation levels after DMSO, HDACi, PKCa or aCD3/CD28 treatment in CD4 T cells with dimensionality reduction by tSNE showed clustering of DMSO and HDACi treatment while PKCa and aCD3/28 clustered separately (Fig 1E), in accordance with the decreased peptidase activities induced by HDACi and the increased peptidase activities induced by PKCa.

### The changes in cytosolic peptidase activities following LRA treatment alter antigen processing

**Fig 2:**
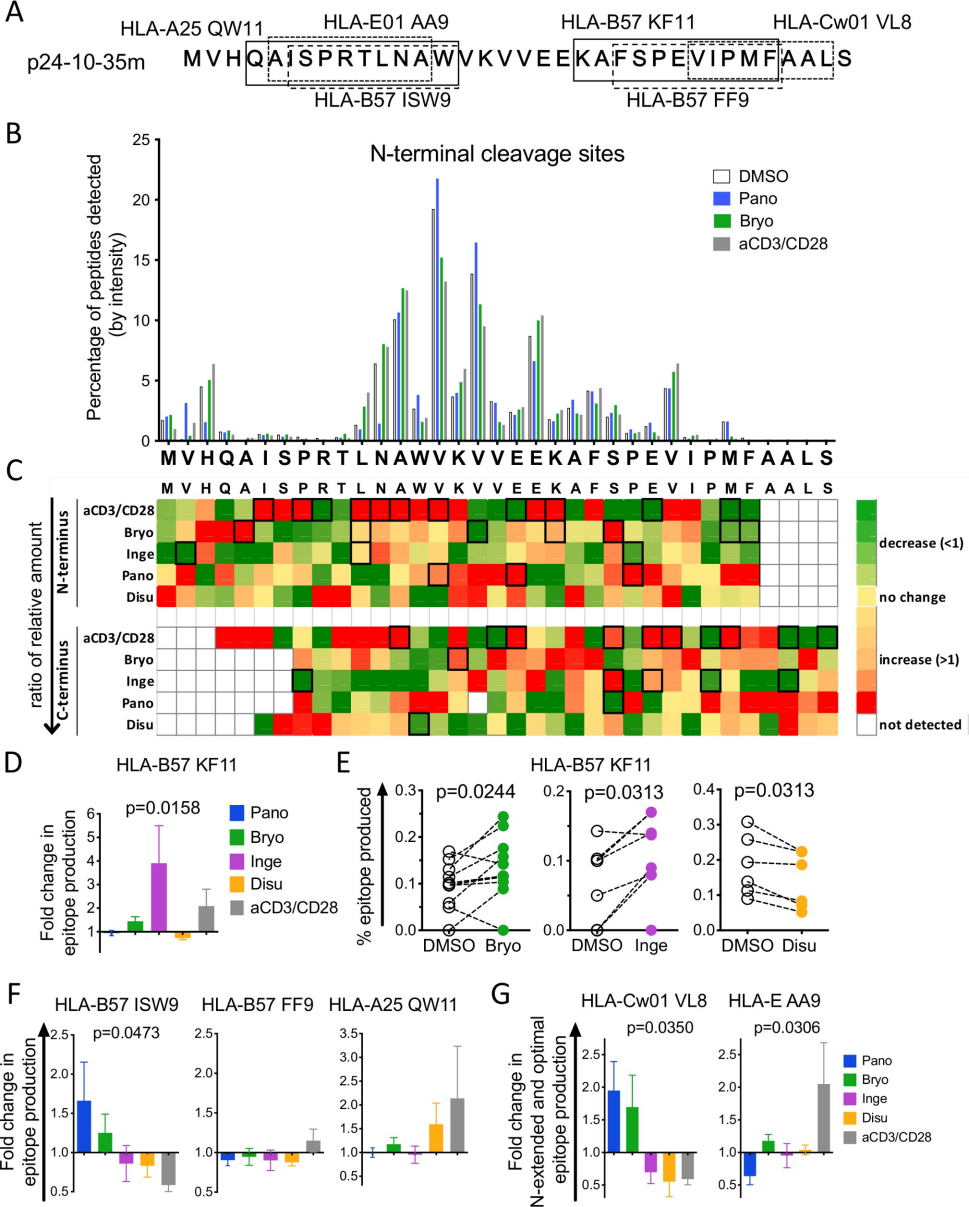
LRA treatments alter the degradation pattern of HIV-1 p24-10-35m and epitope production. A. Sequence of HXB2 HIV-1 Gag p24-10-35m substrate and MHC-I optimal epitopes. B. Cleavage patterns of p24-10-35m after a 120-minute degradation in extracts of primary CD4 T cells treated with DMSO- (open bars), Panobinostat- (blue), Bryostatin- (green) or aCD3/CD28–treated (grey) showing the relative amount of fragments starting at each residue (N-terminus cleavage site). Results shown for a representative experiment. C. Ratios of the relative amount of fragments starting (top) and ending (bottom) at each residue in matched DMSO- over LRA-treated samples (n = 6–15). Changes in fragments starting (N-terminus) or ending (C-terminus) at each position appear as decreased (green; <1), increased (orange/red; >1) or unchanged (yellow) compared to DMSO-treated samples. Wilcoxon matched-pairs signed-rank t tests were performed on the matched DMSO- and LRA-treated samples. The boxed squares indicate statistical significance (p < 0.05). D. Fold change in HLA-B57-KF11 epitope production in CD4 T cell extracts treated with Panobinostat- (blue), Bryostatin- (green), Ingenol- (purple), Disulfiram- (orange) or aCD3/CD28-treated (grey) extracts over matched DMSO-treated extracts (n = 6–10 donors). One-Way ANOVA Kruskal-Wallis tests on the 5 groups and the p-value is reported. E. Percentage of HLA-B57-restricted KF11 epitope produced in paired DMSO- (open circles) and Bryostatin- (green, n = 9, left), Ingenol- (purple, n = 6, middle) or Disulfiram- (orange, n = 6, right). Wilcoxon matched-pairs signed-rank t tests were performed. F. Fold change in HLA-B57 ISW9 (left, n = 4–9 donors), HLA-B57 FF9 (middle, n = 5–12 donors) and HLA-A25 QW11 (right, n = 2–6 donors) epitope production in paired Panobinostat- (blue), Bryostatin- (green), Ingenol- (purple), Disulfiram- (orange) or aCD3/CD28-treated (grey) CD4 T cell over paired DMSO-treated extracts. G. Fold change in HLA-Cw01 VL8 (left, n = 2–6 donors) and HLA-E01 AA9 (right, n = 5–9 donors) epitope and N-extended precursors production in Panobinostat- (blue), Bryostatin- (green), Ingenol- (purple), Disulfiram- (orange) or aCD3/CD28-treated (grey) CD4 T cell extracts over paired DMSO-treated extracts. F and G: One-Way ANOVA Kruskal-Wallis tests were performed on the 5 groups and the p-value is reported if statistically significant.

**Fig 3:**
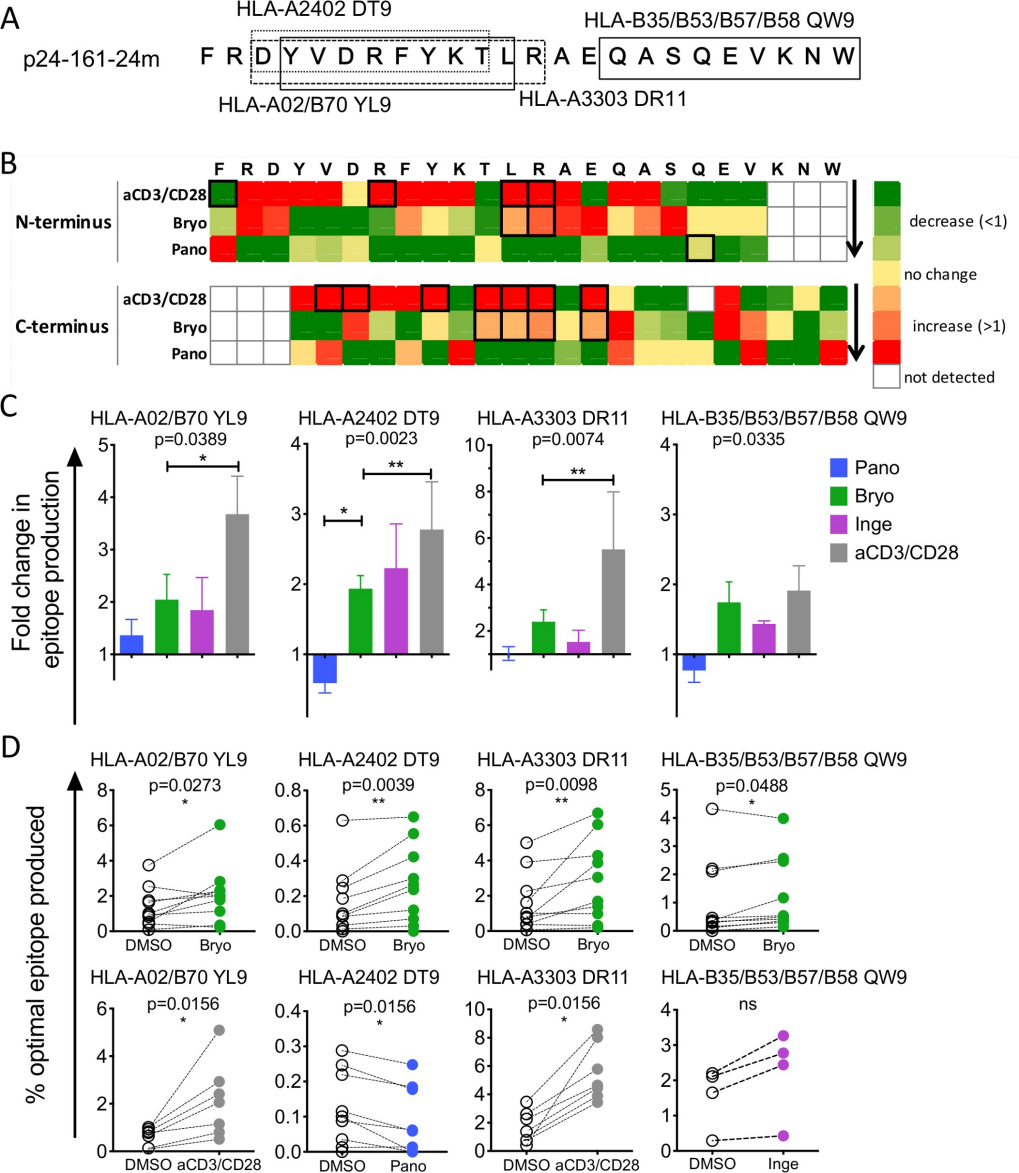
LRA treatments alter the degradation patterns of HIV-1 p24-161-24m and epitope production. A. Sequence of HXB2 HIV-1 Gag p24-161-24m and MHC-I HIV epitopes. B. Reading vertically the heat map shows changes in the amount of degradation fragments starting (N-terminus, top) or ending (C-terminus, bottom) at each position upon LRA treatment over matched DMSO-treated sample in an average of n = 8–11 healthy donors. Wilcoxon matched-pairs signed-rank t tests were performed on matched DMSO- and aCD3/CD28- or LRA-treated primary CD4 T cells extracts. The boxed squares indicate statistical significance (p < 0.05). C. Fold change in HLA-A02/B70 YL9 (left, n = 5–10 donors), HLA-A2402 DT9 (middle left, n = 5–12 donors) and HLA-A25 QW11 (right, n = 2–6 donors) epitope production in Panobinostat- (blue), Bryostatin- (green), Ingenol- (purple), or aCD3/CD28-treated (grey) CD4 T cell extracts over paired DMSO-treated extracts. One-Way ANOVA Kruskal-Wallis tests were performed on the 4 groups and the associated p-value is reported, Dunn’s Multi-comparison test was calculated and the pairs that statistically differed are indicated with a bar. D. The percentage of HLA-A02/B70 YL9, HLA-A2402 DT9, HLA-A3303 DR11 and HLA-B35/B53/B57/B58 QW9 epitopes produced in paired DMSO- (open circles) and Bryostatin- (green, n = 8–10), Ingenol- (purple, n = 4), Panobinostat- (blue, n = 9) or aCD3/CD28-treated (grey, n = 7) extracts. Wilcoxon matched-pairs signed-rank t tests were performed. * p < 0.05, ** p < 0.01, ns: not significant.

We evaluated if the changes in cellular peptidase activities induced by LRAs affected the degradation of two long HIV peptides and the production of HIV epitopes (Figs 2 and 3). First, we compared the degradation of a HIV-1 Gag 35mer containing 6 MHC-I-restricted HIV epitopes, p24-10-35m MVHQAISPRTLNAWVKVVEEKAFSPEVIPMFAALS, in matched cellular extracts of primary CD4 T cells treated for 48 hours with DMSO, aCD3/CD28, Bryostatin, Ingenol, Panobinostat or Disulfiram (Fig 2A). Degradation peptides generated at various time points were purified, identified and quantified by mass spectrometry as in [37]. We compared the degradation patterns of the 35-mer by quantifying the relative amount of peptides starting or ending at each aa. A representative diagram of the N-terminal cleavage sites is shown in Fig 2B. While the main cleavage sites (H3, A13, V15, V17, E20, V27) were similar in the DMSO-, LRAs- and aCD3/CD28-treated cells, the amount of fragments starting at each residue varied with the treatment. For instance, fragments starting at H3 or N12 were reduced by 10-fold in Panobinostat-treated extracts, while those starting at V2, V15, V17 were increased by Panobinostat and reduced by Bryostatin and aCD3/CD28. To measure the changes in cleavage sites in multiple donors (n = 6–15), we calculated the average fold change at each aa (peptide amount obtained for LRA-treated divided by matched DMSO-treated extracts), and plotted the values as heatmaps for N-terminal (top) and C-terminal (bottom) cleavage sites (Fig 2C; compare intensity of each aa vertically). Most cleavage sites were variably increased or decreased by treatments. Anti-CD3/CD28-stimulation caused the most frequent and significant changes with 12 altered cleavage sites at the N-terminus (9 increased, 3 decreased) and 10 positions for the C-terminus (6 increased, 4 decreased), in accordance with our published results [37]. PKCa treatment significantly changed several N- and C-terminus cleavage sites but distinct from aCD3/CD28-induced changes. Ensuing the opposite changes in peptidase activities, HDACi and PKCa had opposite effects on specific cleavage sites (for instance more peptides starting at V17 in Panobinostat and fewer in Bryostatin-treated extracts).

We then assessed the production of known HIV-1 CTL epitopes spanning p24-10-35m (Fig 2A and 2C). Out of 11 known HIV epitopes 10 were produced as optimal or N-extended peptides across 20 donors. We calculated the fold change in epitope production by dividing the relative amount of epitope produced in LRA-treated extracts by the value obtained for the matched DMSO control across all experiments (Fig 2D and 2E). HLA-B57 KF11 production was increased by PKCa and aCD3/CD28, unchanged by Panobinostat and decreased by disulfiram (Fig 2D). Since the impact of LRA treatment is variable across donors (as seen for all HIV-related parameters measured ex vivo or in vivo on LRA-treated cells [14], we plotted the amount of KF11 produced in paired DMSO/LRA samples, showing significantly increased KF11 production in PKCa-treated extracts and reduced KF11 production in disulfiram-treated extracts in most donors (Fig 2E). The production of 3 other HLA-B57 HIV epitopes (QW11, ISW9 and FF9) was variably affected by LRA treatments (Fig 2F). HLA-B57 FF9 was mostly unchanged by all treatments while QW11 production was increased in aCD3/CD28- or disulfiram-treated extracts. Interestingly ISW9 was the only epitope increased by Panobinostat treatment (low peptidase activities) and decreased by aCD3/CD28 treatment. Epitopes produced mostly as shortly N-extended peptides such as Cw-01 VL8 and HLA-E AA9 also showed different production patterns according to LRA treatments. Like ISW9, VL8-containing peptides accumulated with treatments lowering peptidase activities while AA9 production was only increased in aCD3/CD28 extracts (Fig 2G).

We similarly assessed the processing of another HIV fragment, p24-161-24mer, after LRAs or aCD3/CD28 treatments (Fig 3A). Anti-CD3/CD28 treatment changed 12 (11/12 increased) N- or C-terminal cleavage sites while Bryostatin treatment significantly increased 6 cleavage sites. Panobinostat decreased peptide production at most aa (Fig 3B). The production of epitopes HLA-A02 YL9, HLA-A24 DT9, HLA-A33 DR11 and HLA-B35 QW9 were increased in PKCa- and aCD3/CD28-treated extracts (Fig 3C) with variable fold change across donors in LRA-treated extracts (Fig 3D). Altogether these data show that HDACi and PKCa induced heterogenous and often opposite changes in cleavage sites which variably affected HIV epitope production. While many known HIV epitopes saw increased production upon PKCa treatment and reduction or limited changes upon HDACi treatment, other epitopes such as B57-ISW9 or CW01-VL8 were increased in conditions lowering peptidase activities (HDACi treatment). We previously showed ISW9 is among the most degradable p24 sequences while KF11 or DT9 were less degradable in the cytosol of primary cells [51]. Thus, the degradability of a specific sequence together with drug-induced increased or decreased hydrolytic activities will set changes in peptide production upon LRA treatment. Highly degradable sequences may only accumulate in reduced hydrolytic environment while poorly degradable epitopes may be processed faster in higher hydrolytic conditions.

### LRAs change the production of degradation peptides and their distribution across the HIV sequence

**Fig 4:**
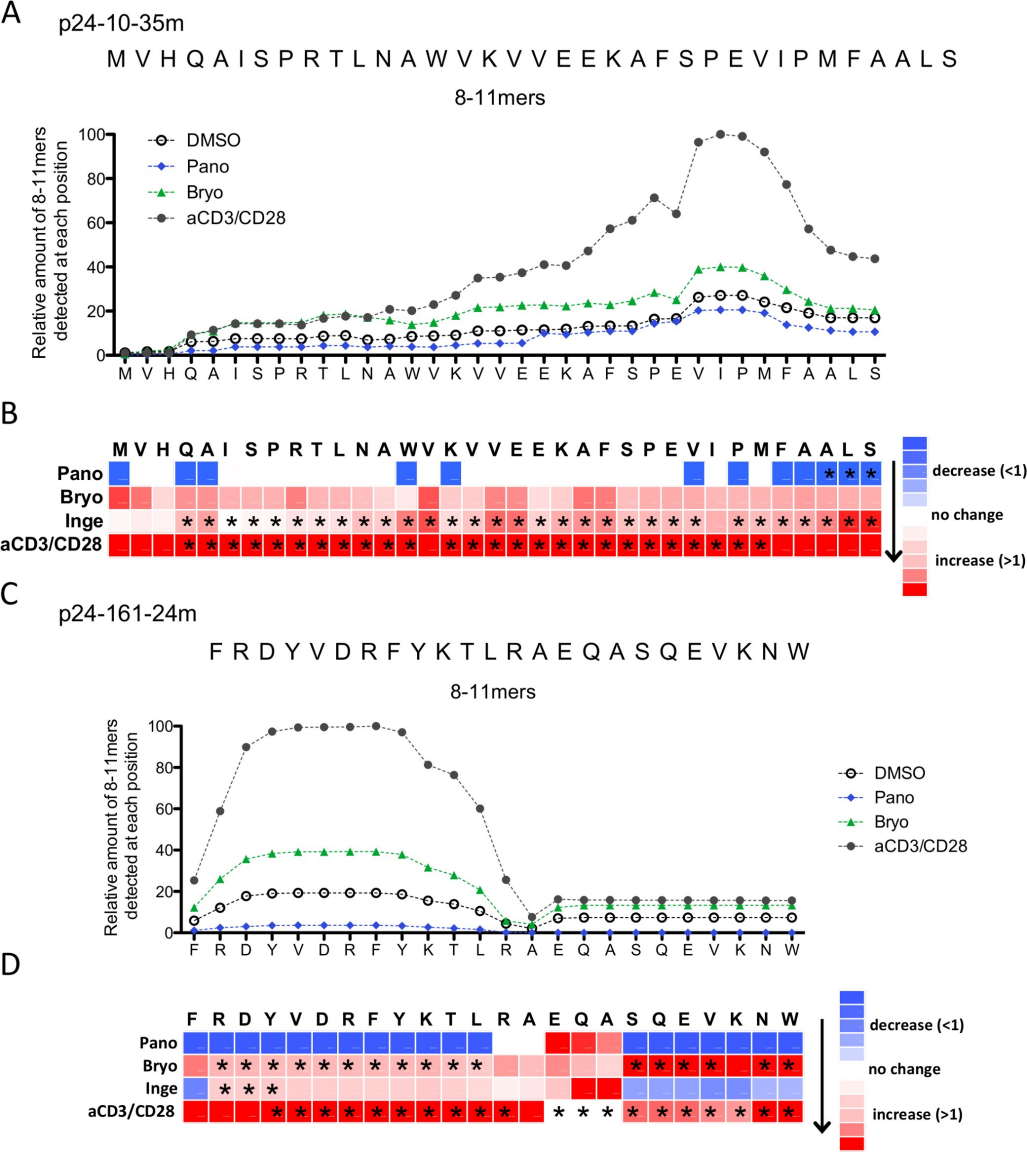
LRA treatments alter the degradation patterns of HIV-1 long peptides into peptides of 8-11aa beyond known HIV epitopes. A. Relative amount of degradation peptides of 8-11aa detected at each residue of p24-10-35m during degradation in CD4 T cell extracts treated with DMSO (open circles), Bryostatin (green), Panobinostat (blue) or aCD3/CD28 (grey). Results shown for a representative experiment. B. Heat map showing the changes in the contribution of each residue to the production of 8-11aa peptides during the degradation of p24-10-35m in Panobinostat, Bryostatin, Ingenol or aCD3/CD28 over matched DMSO control. n = 7–11 donors. Stars show statistically significant changes (p<0.05). C. Same as A for p24-161-24m degradation peptides. D. Heat map similar to B for p24-161-24mer. n = 4–10 donors.

We analyzed whether LRA-induced changes in peptidase activities and cleavage sites could broaden or narrow the production of degradation peptides of 8-11aa (a size compatible with MHC-I loading) beyond known HIV epitopes (Fig 4). For both p24-10-35m and p24-161-24m we measured the contribution of each aa to the amount of 8-11aa peptides across the sequence, generating a density plot (Fig 4A and 4C) and a heatmap (Fig 4B and 4D) of the sequence coverage by 8-11aa degradation peptides. In p24-10-35m the amount of 8-11aa peptides was higher in Bryostatin- or aCD3/CD28-treated extracts than in DMSO- or Panobinostat-treated extracts, and the difference was heightened by up to 5-times in the second half of the sequence (Fig 4A). This is in agreement with the lower degradability of the second half of p24-10-35m that may subsist under higher hydrolytic activities induced by PKCa or aCD3/CD28 [37,51]. We then calculated a ratio of peptide coverage in treated over DMSO extracts for each condition in multiple donors (n = 7–11) (Fig 4B). The coverage of the sequence by 8-11aa peptides in Panobinostat-treated cells was similar to that of DMSO-treated cells with the exception of 3 residues with significantly lower coverage. In extracts from aCD3/CD28 and from Ingenol-treated cells the coverage of the sequence by 8-11aa was significantly higher throughout most of the 35-mer, in accordance with increased peptidase activities leading to more frequent cleavage events.

In p24-161-24m, Panobinostat treatment reduced the production of 8-11aa peptides throughout the sequence while Bryostatin and aCD3/CD28 increased it in the first half of the sequence by 2- to 5-fold (Fig 4C). The ratio of 8-11aa produced in treated over DMSO treated cells in n = 4–10 donors significantly increased in Bryostatin- and aCD3/CD28-treated cells throughout most of the sequence and only in the first half of the sequence in Ingenol-treated cells (Fig 4D). Throughout both p24 fragments PKCa treatments increased the density of 8-11aa degradation peptides in areas most resistant to complete degradation [36,51], providing a possible approach to increase production of nested peptide sets for MHC presentation.

### Combination treatment of HDACi and PKCa modulate HIV antigen processing

**Fig 5:**
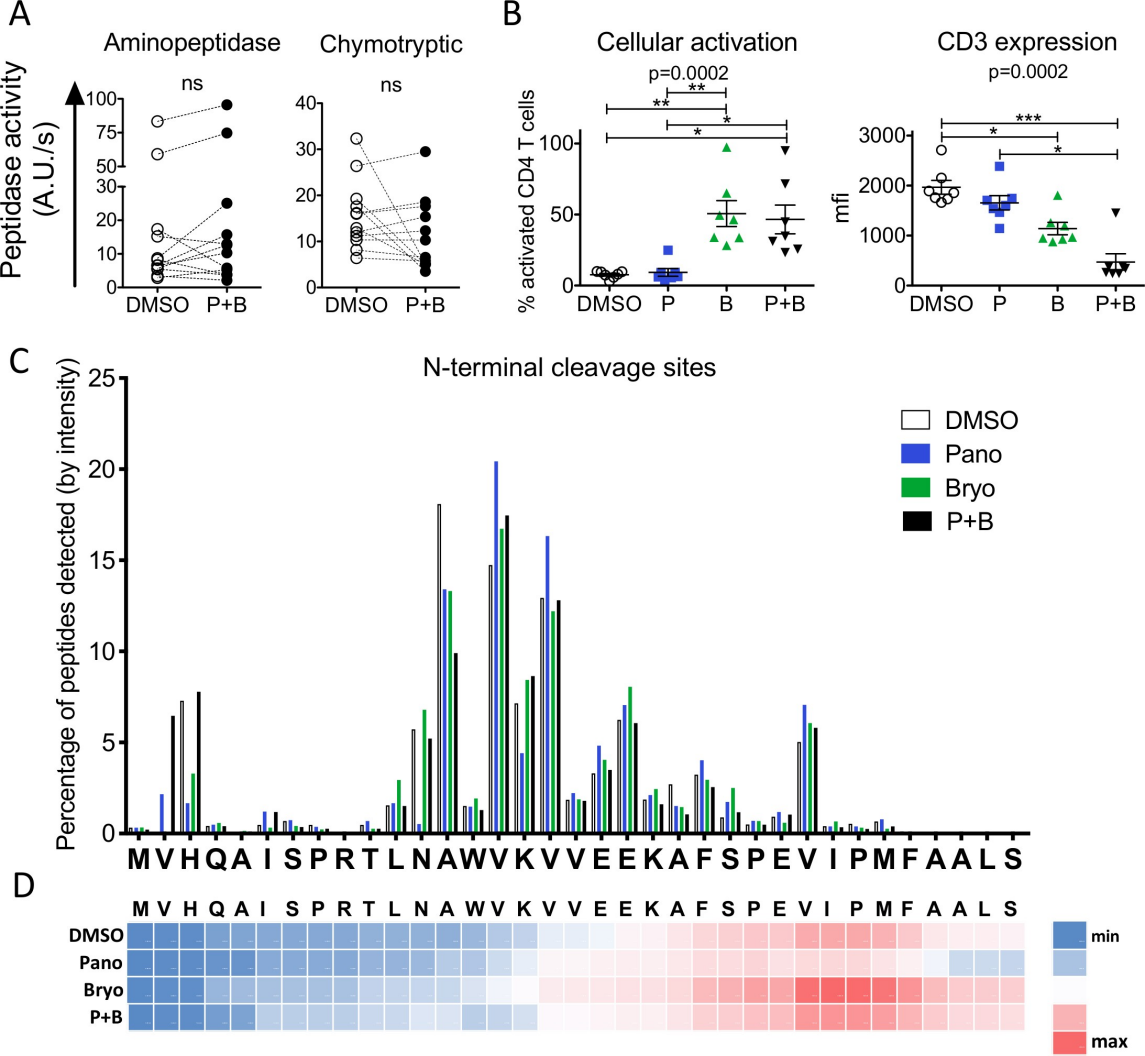
Combined Bryostatin and Panobinostat treatment of CD4 T cells changes HIV peptide degradation patterns. A. Aminopeptidase (left) and proteasomal chymotryptic (right) hydrolytic activities in paired DMSO- (open circles) and Panobinostat+Bryostatin-treated (filled circles) primary CD4 T cells measured at 48 h post-treatment. n = 12 healthy donors. Wilcoxon matched-pairs signed-rank t tests. ns: not significant. B. The surface expression of CD3 (right) and CD25 and CD69 (left) was monitored by flow cytometry in primary CD4 T cells at 48 h post treatment with DMSO (open circles), Panobinostat (blue squares), Bryostatin (green triangles), a combination of Panobinostat+Bryostatin (black inverted triangles) (n = 7 healthy donors). One-Way ANOVA Kruskal-Wallis tests were performed on the 4 groups and the overall p-value is reported, Dunn’s Multi-comparison test was calculated and the pairs that statistically differed are indicated with a bar. * p < 0.05, ** p < 0.01, *** p < 0.001. C. Cleavage patterns of p24-10-35m after degradation in extracts from DMSO- (open bars), Panobinostat- (blue), Bryostatin- (green) and combination Panobinostat+Bryostatin-treated (black) primary CD4 T cells extracts at 120 min, showing the relative amount of fragments starting (N-terminus cleavage site) at each residue. Results shown for a representative experiment. D. Heat map representing the relative amount of 8–11 aa peptides containing each aa. Each row represents an LRA-treatment. One representative experiment is shown.

Since combinations of HDACi and PKCa improve HIV provirus reactivation [14,52,53], we assessed the effects of Panobinostat and Bryostatin combination on antigen processing (Fig 5). While treatment with Panobinostat or Bryostatin alone produced opposite effects on hydrolytic activities, the combination treatment did not significantly change -on average- aminopeptidase and proteasomal hydrolytic activities compared to DMSO-treated cells. However, we observed large differences across donors in agreement with the variable effects of LRAs across donors [14,52–55]. Interestingly the combination treatment did not reduce cellular activation compared to Bryostatin treatment (Fig 5B left panel). Treatment of primary CD4 T cells with either drug or even more for the HDACi+PKCa combination reduced CD3 expression (Fig 5B right panel) as well as CD4 surface expression.

We compared the effect of single LRA or combination treatment on the degradation of HIV p24-10-35m (Fig 5C). While the 3 main cleavage sites occurred at the same residues in all conditions (A13, V15, V17), the relative abundance of peptides varied. Peptide abundance in combination treatment sometime followed that of Bryostatin-treated cells (N11, V15, V17) and less frequently that of Panobinostat-treated cells (I6). The combination treatment had additive effect in decreasing peptide production (A13), increasing others (V2) or restored the production of peptides (H3). Coverage of an antigenic fragment by 8-11aa degradation peptides can be modulated and was intermediate between Bryostatin- or DMSO-treated extracts after HDACi+PKCa combination treatments (Fig 5D).

### LRA treatments affect endogenous processing and presentation of HIV epitopes to CD8 T cells

**Fig 6:**
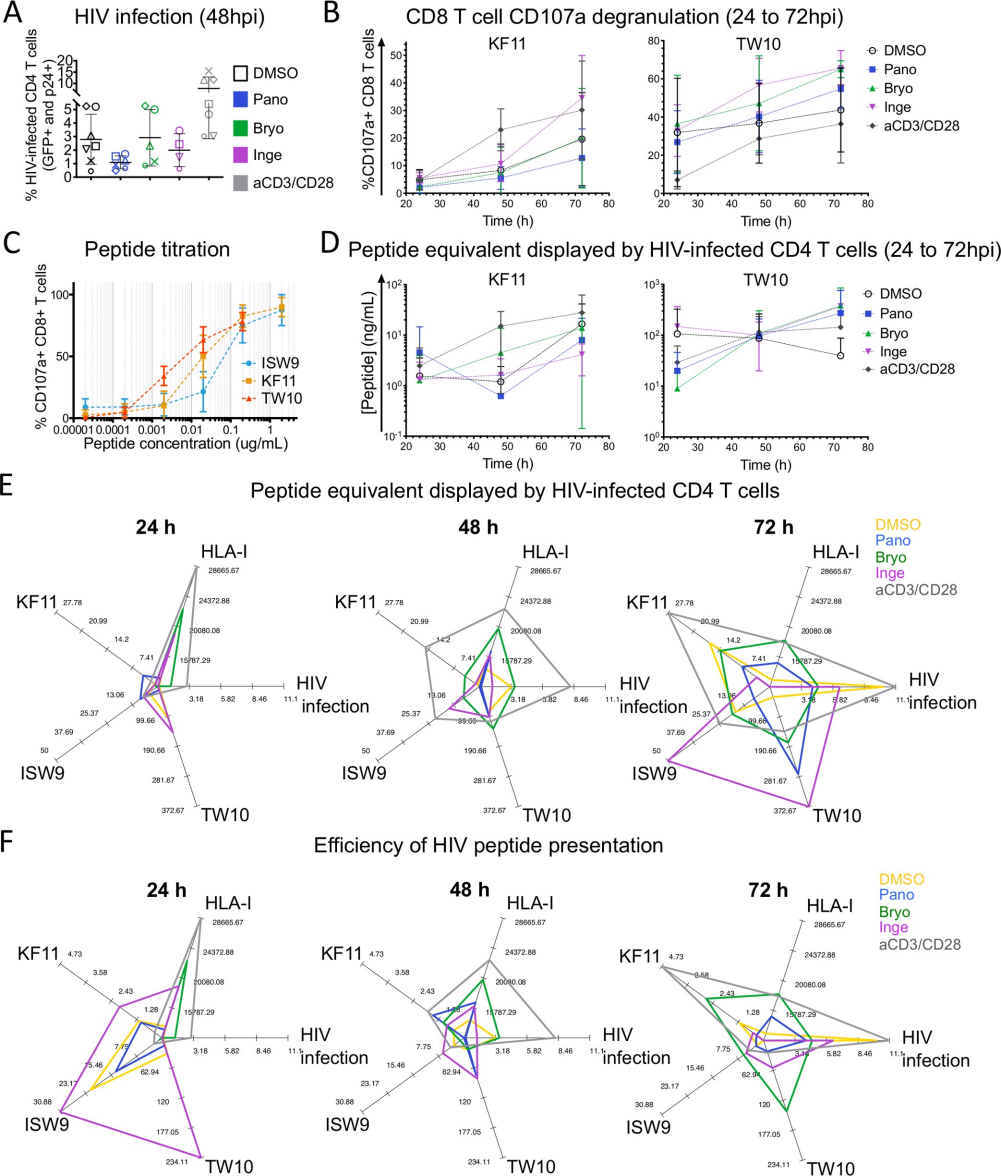
LRAs affect endogenous HIV epitope processing and presentation by HIV-infected CD4 T cells to CD8+ T cells. A. HIV-1ΔEnv-GFP-VSVg infection rate of primary CD4 T cells pretreated with DMSO (black), aCD3/CD28 (grey) or Panobinostat (blue), Bryostatin (green), Ingenol (purple) was assessed by flow cytometry using GFP and HIV-p24 expression at 48 h post-infection (n = 7 donors with distinct symbols). B. Percentage of CD107a+ CD8 T cells specific for HLA-B57 KF11 (left) and HLA-B57 TW10 (right) after incubation with HLA-B57+ HIV-infected CD4 T cells treated with DMSO, LRA or aCD3/CD28 at 24, 48 and 72h post-infection at a 4:1 CTL:CD4 ratio. For each treatment (DMSO, LRA or aCD3/CD28 beads) control uninfected LRA- or beads-treated CD4 T cells were included in the experiment and used to calculate and subtract CD107a background. C. Percentage of CD107a+ CD8 T cells specific for HLA-B57 ISW9- (cyan), KF11- (orange) and TW10- specific (red) after incubation with peptide-pulsed DMSO-treated HLA-B57+ CD4 T cells measured at a 4:1 CTL:CD4 ratio. A-C: n = 6 experiments with mean values and standard deviations. D. Peptide equivalent (KF11 (right) and TW10 (left), displayed at the surface of HIV-infected HLA-B57+ CD4 T cells treated with DMSO (open blank symbols), Panobinostat (blue squares), Bryostatin (green triangles), Ingenol (inverted purple triangles) and CD3/CD28-stimulated (grey diamonds). The results of n = 5 (TW10) and n = 4 (KF11) infection and degranulation experiments are shown at 24, 48 and 72 hpi. E. Spider plots showing the peptide equivalent (TW10, KF11, ISW9 in ng/ml), HIV infection rates (% GFP^+^p24^+^), MHC-I surface levels (mfi) displayed by HIV-infected CD4 T cells pre-treated with DMSO (yellow), aCD3/CD28 (grey), Panobinostat (blue), Bryostatin (green), Ingenol (purple). Axes for any given parameter were scaled uniformly across timepoints to take into account the full dynamic range (i.e., minimum to maximum value for that parameter across the 3 timepoints). Spider plots at 24, 48 and 72 h (left to right) after HIV infection of pre-treated CD4 T cells. F. Spider plots similar to E showing the efficiency of peptide presentation (TW10, KF11, ISW9) defined at the peptide equivalent (TW10, KF11, ISW9) displayed by HIV-infected CD4 T cells divided by the infection rate (% GFP^+^p24^+^, an indirect measurement of HIV antigens in cells).

To determine if LRA-induced changes in epitope production measured in vitro reflect changes in endogenous processing and presentation, we measured epitope-specific CTL activation by HIV-infected CD4 T cells pretreated with LRAs (Fig 6). LRA-treated CD4 T cells were infected with single round HIV-1Δenv expressing GFP and pseudotyped with VSVg envelope. HIV infection, MHC-I levels and recognition by CTL specific for 3 HIV-1 Gag p24 epitopes were assessed at 24, 48 and 72 hours post-infection.

We first assessed whether LRA treatments affect HIV infection of primary CD4 T cells as it contributes to the amount of antigen available for processing (Fig 6A showing 48 hpi). As previously observed HIV infection of resting CD4 T cells was lower than that of aCD3/CD28-activated CD4 T cells (3.1 vs. 6.6% GFP^+^p24^+^ positive cells, respectively) with expected variability across donors. In contrast the infection rates of LRA-treated cells did not follow LRA-induced activation levels. HDACi (no cellular activation) pre-treatment reduced infection by 3-fold compared to resting CD4 T cells while PKCa-treated cells triggering intermediate or high activation levels had infection rates below or similar to resting CD4 T cells at 48 hpi, well below CD3/CD28-activated cells. The kinetics of HIV infection between 24 to 72 hours remained slower and lower in LRA-treated cells than in untreated or TCR-activated CD4 T cells (S2A Fig). MHC-I levels were higher in the presence of PKCa or aCD3/CD28 on the day of infection and decreased over 72 hours back to control levels (S2B Fig).

We measured CD107a degranulation of CD8 T cells specific for HLA-B57-restricted HIV-1 Gag p24 KF11, TW10 and ISW9 over 72 hours post-infection. CD107a degranulation by KF11-specific CTL (Fig 6B) and ISW9-specific CTL (S2C Fig) were similar or lower in the presence of Panobinostat-treated or Bryostatin-treated HIV-infected CD4 T cells than in DMSO-treated cells. Only Ingenol-treated cells yielded CD107a similar to aCD3/CD28-stimulated cells at 72 hpi. In contrast CD107a degranulation of TW10-specific CTL was the highest of the 3 clones in the presence of infected cells treated with any LRA and became higher than with aCD3/CD28-treated cells at 48-72hpi. HLA-B57 TW10 epitope which has the advantage of being less degradable [37,51], presentable as both optimal and N-extended peptides [56], and having high affinity for MHC and TCR efficiently activated CTL.

CD8 T cells activation depends on the functional avidity of the CTL and on the amount of cognate peptide endogenously processed and presented by HIV-infected CD4 T cells. To define CTL functional avidity, we measured CTL degranulation in the presence of CD4 T cells exogenously pulsed with increasing amount of each peptide, ISW9, KF11 and TW10 (Fig 6C). The peptide titration of the three CTL clones showed a lower avidity of ISW9-specific CTL, intermediate for KF11 and higher for TW10 in accordance with the higher HLA-B57 binding affinity of KF11 and TW10 compared to ISW9 [37,51,57] (Fig 6C). We estimated the relative amount of each peptide presented by infected cells. We calculated an equivalent amount of peptide displayed by HIV-infected CD4 T cells by comparing the percentage of degranulation elicited by incubation of CTL with HIV-infected cells pre-treated with various LRAs to the % obtained with CD4 T cells pulsed with increasing amounts of peptide (Fig 6D). KF11 peptide equivalent displayed by HIV-infected CD4 T cells and ISW9 peptide presentation (S2D Fig) showed similar pattern with a reduction of peptide presentation in Panobinostat-treated cells at 48 hours and a time-dependent increase in Ingenol- and CD3/CD28-activated cells. TW10 peptide presentation was the highest of all 3 epitopes and moderately affected by treatments (99 to 114 ng/ml compared to 97 ng/ml in DMSO-treated cells). These data emphasized the uneven time-dependent processing and presentation of different peptides within the same HIV protein in HIV-infected CD4 T cells [37] and showed a variable effect of LRAs on peptide presentation according to epitopes as shown in the in vitro epitope processing experiments.

During viral infection peptide display by MHC-I is variable and dynamic [37,58–60] as it may be altered by variations in amount of antigens, MHC-I or antigen processing activities. Radar plots at 24, 48 and 72 hpi showed the equivalent TW10, ISW9 and KF11 peptides displayed by HIV-infected CD4 T cells along the infection rates (GFP+p24+) and MHC-I levels of the same LRA-treated HIV-infected CD4 T cells scaled to the maximum value of each parameter across time points (Fig 6E). At 24 hpi the equivalent peptide presented was the lowest of all three time points for all 3 peptides despite presenting the highest level of MHC-I. Despite low levels of HIV p24 and MHC-I, Panobinostat-treated cells (blue line) had the highest KF11 and ISW9 peptide presentations at 24 hours. In contrast despite having the second highest levels of MHC-I and HIV infection the amount of ISW9, KF11 and TW10 presented by Bryostatin-treated cells (green line) and aCD3/CD28-activated cells (grey line) were among the lowest. At 48 hours aCD3/CD28-activated cells (grey line) had the highest equivalent peptide display for all 3 epitopes. At 72 hours when HIV infection rates were the highest, Ingenol-treated cells had the highest ISW9 and TW10 peptide equivalent presentation despite displaying the lowest levels of MHC-I. Altogether the data show that the equivalent HIV peptide presentation varied over time and was variably affected by LRAs but was not directly correlated with MHC-I levels or infection rates.

Considering the impact of LRAs on the kinetics of HIV antigen expression (S2A Fig) we calculated a score of efficiency of HIV peptide presentation defined as the ratio of peptide equivalent displayed by CD4 T cells over the HIV infection rates (GFP^+^p24^+^), an indirect measurement of the amount of HIV antigens present inside cells (Fig 6F). At 24 hours Ingenol-treated cells had the highest score of peptide presentation efficiency for all 3 epitopes. DMSO-treated and Panobinostat-treated cells were the second highest. The efficiency of presentation was the lowest for aCD3/CD28- and Bryostatin-treated cells. Overtime the efficiency of peptide presentation by Ingenol-treated or DMSO-treated cells decreased while that of aCD3/CD28- and Bryostatin-treated cells increased. The data showed a dynamic peptide presentation not solely driven by variations in MHC-I or antigen levels. Additionally, they unexpectedly revealed that LRAs such as Ingenol can increase at early time points the efficiency of HIV peptide presentation despite low levels of antigens.

## Discussion

The underlying principle of shock and kill strategies is to unlock HIV transcriptional blocks with latency reversal agents, induce HIV expression and cell death by apoptosis [3] or immune clearance [2,3]. LRAs tested so far induced HIV RNA expression to various extents but led to relatively small changes in the size of reservoirs in vivo [9,61]. Beside the efficacy of LRAs, the capacity of pre-existing immune responses to clear reservoirs is uncertain and additional vaccine-induced immune responses are likely needed [10,11,13,62,63].

Most LRAs selected so far to relieve transcriptional blocks act on signaling pathways and histone acetylation [5,64] but are not specific to HIV LTR promoters or targeted to the reservoirs, thus their effect should be considered globally on genes and cellular machineries of cell populations [48–50,65]. The LRA-induced changes in cellular peptidase activities took 48 hours to reach maximum effect both for HDACi and PKCa, suggesting a transcriptional effect either on the expression of the antigen processing machinery or on proteins regulating its activity rather than a direct binding to peptidases’ catalytic sites. Bryostatin can enhance proteasome activities in fibroblasts [43] while disulfiram effect on NF-κB inhibited proteasome activities in cell lines [42]. The RNA expression of some proteasome subunits was modulated in an in vitro latency model upon LRA treatment [55] but neither proteasome expression and hydrolytic activities, nor the impact on antigen processing were assessed.

LRA-induced changes in peptidase activities of CD4 T cells altered degradation patterns of long HIV peptides and epitope production in a sequence- and time-dependent manner. To explain the heterogenous effect of LRA on peptide production and presentation, one needs to consider two interconnected parameters: 1) the intracellular hydrolytic activity levels that are defined by the cell type and the treatment applied to cells, and 2) the degradability of a given antigenic sequence. We and others showed that various CD4+ cell types [35,36,39,40,66] and resting and TCR-activated CD4 T cells [37] present different levels of cellular peptidase activities that influence HIV degradation patterns. Additionally HIV sequences within a protein are variably sensitive to cytosolic degradation, as illustrated by the higher degradability of the first half of the p24-10-35mer containing ISW9 epitope compared to the second half of the 35mer containing HLA-B57 KF11. We showed that even peptides of 8-11aa present a cytosolic half-life varying from a few seconds to hours [51]. This variable sensitivity of HIV sequences to cytosolic degradation is driven by motifs associated with sensitivity/resistance to degradation and shapes the amount of peptides available for MHC loading [51]. When peptidase activities are reduced as seen with HDACi treatment, sequences highly sensitive to degradation are less degraded and better presented as seen for ISW9 at early time points. Conversely poorly degradable sequences are processed and trimmed slower, leading to a lesser and/or slower presentation of degradation-resistant peptides and indirectly favoring the early presentation of the most degradable areas of HIV. Upon increased peptidase activities (as seen with PKCa treatment) cutting events and peptide trimming or degradation increase which could accelerate the production of the less degradable peptides such as intracellularly highly stable HLA-B57 TW10 or HLA-B57 KF11 but conversely increase the degradation of the most sensitive sequences such as HLA-B57 ISW9. Since the sensitivity or resistance to degradation is driven by motifs it will eventually be possible to identify and predict areas of HIV antigens that may yield 8-11aa peptides, or conversely define hydrolytic activities yielding the largest number of 8-11aa. The increased coverage of long HIV peptides by degradation products of 8-11aa in the presence of PKCa provides an initial way to increase the variety and/or number of peptides available for MHC presentation.

The endogenous processing and MHC presentation of HIV peptides to immune cells encapsulates not only the kinetics and amount of peptide production but also MHC levels and binding affinity of peptides (optimal epitopes or extended versions) for MHC and the TCR. When accounting for the amount of HIV antigen available inside cells and MHC levels, Ingenol was remarkably efficient at increasing the presentation of all 3 HLA-B57 peptides at early times (when antigen amount was low) while Bryostatin was efficient at late time points. Whether this relative enhanced peptide presentation remains true for other HIV epitopes remains to be determined. However, the increased production of 8-11aa long peptides and the enhanced efficiency of peptide presentation at low levels of antigens induced by certain LRAs offer new opportunities to broaden the MHC-peptide repertoire for immune detection and define novel targets for immune clearance after latency reversal. As many HIV-specific pre-existing T cell responses may express inhibitory markers [8–10,28], additional novel vaccine-induced latency reversal-specific immune responses may be needed to clear reservoirs [67]. Eliciting immune responses against areas of HIV proteome whose processing and presentation is enhanced by LRAs even at low levels of antigens may provide an additional way to improve immune detection of reservoirs. It will be critical to assess whether HIV epitope presentation is modulated by LRAs after latency reversal ex vivo in latently infected CD4 T cells from patients or in reservoirs in vivo. Improved ex vivo latency models [55,68], streamlined ways to generate or expand epitope-specific CTL [69] and the promising use of immunodeficient mice repopulated with HIV-infected patients’ PBMCs [70] or of humanized BLT (bone-marrow-liver-thymus) mice in recent latency reversal studies [30] may allow to test how the modulation of peptide presentation by LRAs affects immune responses and could be exploited for reservoir clearance.

While this study was focused on the presentation of peptides restricted by conventional HLAs and mostly HLA-A or -B, the modulation of peptide processing might also affect presentation by HLA-C or non-conventional HLA-E that can activate HIV-specific cytolytic NK cell functions [71,72]. Although a MHC-dependent role of cytolytic NK cells after latency reversal remains to be established, the effect of LRA on HIV peptide presentation could also be considered and exploited for these immune responses [73].

The HDACi and PKCa tested so far in clinical trials and used in this study are not very efficient at reactivating HIV expression from reservoirs. The most efficient LRAs usually induce cellular activation and the addition of immunomodulatory agents (IMA) such as Ruxolitinib [74,75] have been tested ex vivo but even the combination of LRA and IMA modulates HIV antigen processing. New LRAs such as those triggering the non-canonical NF-κB pathway [30] or new LRAs combined with inclusion of TLR ligands [24] or PD-1 blockade [25] will be needed to specifically and efficiently reactivate CD4 cell subsets harboring latent proviruses [55,76]. The present study provides a proof of principle that LRAs, current and future more efficacious drugs, should be assessed not only for their capacity to reactivate HIV transcription or to produce HIV particles in ex vivo assays, but also for their impact on antigen processing and their capacity to promote HIV peptide presentation. Ultimately to eliminate HIV reservoirs once provirus transcription is reactivated, a high expression of antigens is probably not necessary as only a few to a few hundreds of MHC-peptides are needed for T cell-mediated immune recognition. However, enhancing peptide presentation despite low levels of antigens after LRA treatment (rather than aiming for high levels of antigens) and possibly broadening the repertoire of surface peptides provides an initial yet unexplored way toward improved reservoir detection. Predicting, ensuring and even improving the presentation of diverse HIV peptides against which functional pre-existing or new vaccine-induced immune responses will support efficient immune clearance of reservoirs. Beyond HIV reservoir clearance, the capacity of LRAs to modulate the relative presentation of various antigenic fragments and to enhance peptide presentation at low levels of antigens could be exploited and integrated in novel immunotherapy strategies (provided adequate targeting) to clear diseased cells in tumors or other chronic disorders [77].

## Material and methods

### Ethics statement

Cells were isolated from buffy coats of anonymous healthy blood donors obtained from the Massachusetts General Hospital blood center (Boston, MA) after approval by the Partners Human Research Committee under protocol 2005P001218.

### Latency reversing agents

Romidepsin, Bryostatin, Disulfiram (Sigma), Ingenol-3-angelate (Santa Cruz Biotechnologies), Panobinostat (Selleckchem) were dissolved in DMSO (Fisher Scientific) to make stock solutions. Fresh aliquots were used for each experiment. The final concentrations for each LRA in cell culture were as follows: Panobinostat (30 nM), Romidepsin (40 nM), Disulfiram (500 nM), Bryostatin (10 nM), Ingenol-3-angelate (50 nM). Maximum *in vitro* stimulation was achieved by addition of anti-CD3/CD28 magnetic beads (Fisher Scientific) at a ratio of 1:1 bead to T cell as in [37]. For combination treatment, both drugs were added at the same time.

### Primary cells

CD4 T cells were isolated by negative selection from fresh PBMCs purified from buffy coats using a CD4+ T cell enrichment magnetic isolation kit (Stemcell Technologies). CD4 T cells were cultured in the presence of 50 Units/mL of Interleukin-2 (NIH AIDS Research and Reference Reagents Program or R&D systems). Cytotoxic CD8 T cell clones and CD4 T cell clones were isolated by limited dilution, maintained in R10+ medium supplemented with IL-2 and stimulated for proliferation with irradiated PBMCs and OKT3 and CD28.2 or OKT3 antibodies (eBioscience), respectively.

### Peptidase activities in live CD4 T cells

The proteasome hydrolytic activities caspase-like (50 μM Z-LLE-AMC; EMD Millipore), tryptic (50 μM Boc-LRR-AMC; Bachem), chymotryptic (50 μM Suc-LLVY-AMC; Bachem), post-proteasomal aminopeptidases (50 μM H-Leu-AMC; Bachem) were measured in 25x10^3^ live CD4 T cells at given time points after drug treatment with peptidase-specific fluorogenic substrates as in [37,40]. The specificity of each catalytic reaction was checked by pre-incubating cells for 30 min with inhibitors of proteasome (10 μM MG132; Enzo Life Sciences), aminopeptidases (120 μM Bestatin; Sigma-Aldrich), before the addition of substrate. The rate of fluorescence emission, which is proportional to the proteolytic activity, was measured every 600 s at 37°C in a Victor-3 Plate Reader (Perkin Elmer), as in [37].

### In vitro degradation experiments and mass spectrometry

2 nmol of pure peptide (MGH peptide core facility or Biosynthesis, Texas) was degraded with 15 ug of CD4 T cell extracts at 37°C in pH7.4 degradation buffer as in [37,40]. Aliquots were taken at various time points and the reaction was stopped by addition of 5% (v/v) of Formic acid (Thermo Scientific) and the degradation products were purified by trichloroacetic acid (Sigma) precipitation (final concentration 5% (v/v)) and identified by in-house mass spectrometry as previously described [37]. Briefly, equal amounts of the purified degradation products were injected into a NanoLC Ultra-HPLC (Eksigent) for salt removal and separation, then online nanosprayed into an LTQ Orbitrap Discovery mass spectrometer (Thermo) for identification. Peptides were separated in a NanoLC column (ChromXP C18, 3 um 120Å; Eksigent) over a gradient of 2–60% buffer B (buffer A: 0.1% (v/v) formic acid in MS-grade water (Fisher Scientific)); buffer B: 0.1% (v/v) formic acid in MS-grade acetonitrile (Fisher Scientific) in 95 min with a conserved flow rate of 250 nl/min. Mass spectra were recorded in the 370–2000 Daltons range. In the tandem mass spectrometry mode, the eight most intense peaks were selected with a window of 1 Dalton and fragmented using helium as collision gas and a voltage of 35 V. Peaks in the mass spectra were searched against the source peptide databases with Proteome Discoverer (version 1.3; Thermo) and quantitatively analyzed. For a given peptide, the integrated area under the peak is proportional to the relative abundance of the peptide in the sample. Each sample was run on the mass spectrometer at least twice.

### Endogenous processing and presentation assay to CD8 T cells

HLA-B57+ primary CD4 T cells were either treated with LRA, stimulated with anti-CD3/CD28 or kept in culture with no stimulation for 48 hours. The cells were thoroughly washed and magnetic anti-CD3/CD28 beads were removed. For each condition, 2 million of CD4 T cells were infected with 20 ug HIV Gag p24 equivalent of NL4-3-Δenv-GFP virus pseudotyped with Vesicular Stomatitis Virus glycoprotein (VSV-g) in the presence of 5ug/mL polybrene (Sigma) by spinfection for 1.5 h at 2000x g as in [37]. At 24, 48 and 72 h post-infection, CD4 T cells were plated with epitope-specific CD8 T cells at a ratio of 1:4 (CD4:CD8) and an aliquot of CD4 T cells was also harvested to measure HIV-1 infection through GFP and HIV-1 p24 intracellular expression, as well as cellular activation and HLA class I surface expression by flow cytometry. After 30 min of co-culture, CD107a-Pe-Cy7 (BD Biosciences) and CD28/CD49d antibodies (BD Biosciences) were added to each well to measure CTL degranulation. After 6 h cells were harvested for surface staining.

The avidity of the CTL clones was assessed by performing a concentration-course titration. Target HLA-B57 CD4 T cells were incubated with increasing concentration of the cognate peptides (0.000002–2 ug/mL) before co-culture with the corresponding CD8 T cells as described above.

### Flow cytometry

Untreated or LRA-treated CD4 T cells were stained with CD4-BV605, CD3-Pacific Blue (BD Biosciences) and HLA-ABC-AlexaFluor700 (Biolegend) to assess purity (95–99%) and CD38-FITC, CD69-PE, CD25-PerCpCy5.5 and HLA-DR-APC (BD Biosciences) to measure surface activation marker levels. Cellular activation levels were calculated by first gating on the double negative population of CD25 and CD69 of the non-treated sample on live, CD4- and CD3-positive cells and applying the same gates to the LRA-treated and CD3/CD28-stimulated samples as done in [37]. A similar gating strategy was applied to determine the proportion of CD38- and HLA-DR-positive CD4 T cells.

For degranulation experiments using HLA-B57-expressing CD4 T cell clone with CD8 T cell clones, the co-cultured cells were stained with CD4-BV605, CD8-APC-Cy7, CD3-Pacific Blue and CD107a-Pe-Cy7 (BD Biosciences). CD107a-positive cells were gated from the live, CD4-negative, CD3- and CD8-positive cells. Non-HIV-infected CD4 T cells pre-treated with DMSO, LRA or aCD3/CD28 with were used to calculate background degranulation at each time point for each experiment. The target CD4 T cells were stained with CD4-BV605, CD3-Pacific Blue, CD25-PerCpCy5.5, CD69-APC (BD Biosciences), and HLA-ABC-AlexaFluor 700 (Biolegend). HIV-1 infection was assessed by GFP expression and HIV-1 p24 intracellular protein staining using KC57-RD1 HIV p24 antibody (Beckman-Coulter). HIV-infected CD4 T cells were gated as live, CD3- and CD4-positive cells that were both expressing GFP and HIV-1 p24. Non-infected CD4 T cells similarly pre-treated were used to calculate background staining. All mean fluorescence intensity (mfi) values were calculated using geometric mean. Samples were run on a BD 4 Laser LSRII flow cytometer and analyzed using FlowJo software (FlowJo, LLC).

### Statistical analysis and data visualization

Experimental data were analyzed using Microsoft Excel and GraphPad Prism version 5.0f (GraphPad Software). Spearman’s rank correlation was used to examine bivariate associations and Wilcoxon signed rank test was used to compare measurements between paired non-treated and LRA-stimulated samples. All p values are two-sided and p values lower than 5% were considered statistically significant.

Dimensionality reduction was performed on multi-parametric datasets using the t-Distributed Stochastic Neighbor Embedding (t-SNE) algorithm on scaled data [78]. t-SNE was run on centered and scaled (i.e., z-scored) data. Parameters critical for antigen presentation were visualized using spider/radar plots. A different radar plot was generated for each timepoint. However, axes for any given parameter were scaled uniformly across timepoints to take into account the full dynamic range (i.e., minimum to maximum value for that parameter) across the 3 timepoints.

## Supporting information

S1 Fig Different classes of LRA alter cytosolic peptidase activities. Aminopeptidase, proteasomal chymotryptic, tryptic-like and caspase-like hydrolytic activities in paired DMSO- (open circles) and LRA-treated (filled circles) primary CD4 T cells measured at 48 h post-treatment. Panobinostat, Romidepsin, Disulfiram, Bryostatin and Ingenol; results are shown for n = 7–15 healthy donors. Wilcoxon matched-pairs signed-rank t tests were performed. * p < 0.05, ** p < 0.01, *** p < 0.001, ns: not significant.
(TIF)

S2 Fig LRA treatment affects endogenous processing and presentation of HIV epitopes by HIV-infected CD4 T cells to CD8+ T cells. A. Primary CD4 T cells were treated with DMSO (open circles), aCD3/CD28 (grey diamonds), Panobinostat (blue squares), Bryostatin (green triangles), Ingenol (purple inverted triangles) 48 h prior to HIV-1 infection with HIV-1 NL4-3-Denv-GFP pseudotyped with VSVg. The HIV infection rate of primary CD4 T cells was assessed by flow cytometry (GFP and HIV-p24 expression) at 24, 48 and 72 h post-infection. B. HLA class I levels were assessed by flow cytometry at 0, 24, 48 and 72 h post-infection in DMSO, aCD3/CD28 and LRA-treated primary CD4 T cells. Changes upon treatment was expressed as a ratio of HLA-ABC expression of LRA- or aCD3/CD28-treated cells by their matching DMSO counterparts. Red line at ratio value = 1. C. The percentage of CD107a+ HLA-B57 ISW9-specific CD8 T after incubation with DMSO (open circles), aCD3/CD28 (grey diamonds), Panobinostat (blue squares), Bryostatin (green triangles), Ingenol (purple inverted triangles) HLA-B57+ CD4 T cells infected with HIV-1 NL4-3-ΔEnv-GFP pseudotyped with VSVg was measured at 24, 48 and 72 h post-infection at a 4:1 CTL:CD4 ratio. For each treatment (DMSO, LRA or aCD3/CD28 beads) control uninfected LRA- or aCD3/CD28 beads-treated CD4 T cells were included in the experiment and used to calculate and subtract CD107a background. D. The ISW9 peptide equivalent displayed by CD4 T cells pre-treated with DMSO, LRA or aCD3/CD28 was shown at 24, 48, 72 hpi E. The efficiency of HIV peptide presentation score at 24, 48 and 72 hpi was calculated for HLA-B57 ISW9 by dividing the relative amount of peptide presented at the cell surface by the % GFP^+^p24^+^ as indirect measurement of HIV antigen content for each treatment condition at 24, 48 and 72 h post-infection. n = 6 experiments as mean values with standard deviation.
(TIF)
