# Supplementary figures and images for: Latency reversal agents modulate HIV antigen processing and presentation to CD8 T cells
Source: PLoS Pathog. 2020 Mar 20;16(3):e1008442. doi: 10.1371/journal.ppat.1008442 (PMC7112239; doi:10.1371/journal.ppat.1008442)

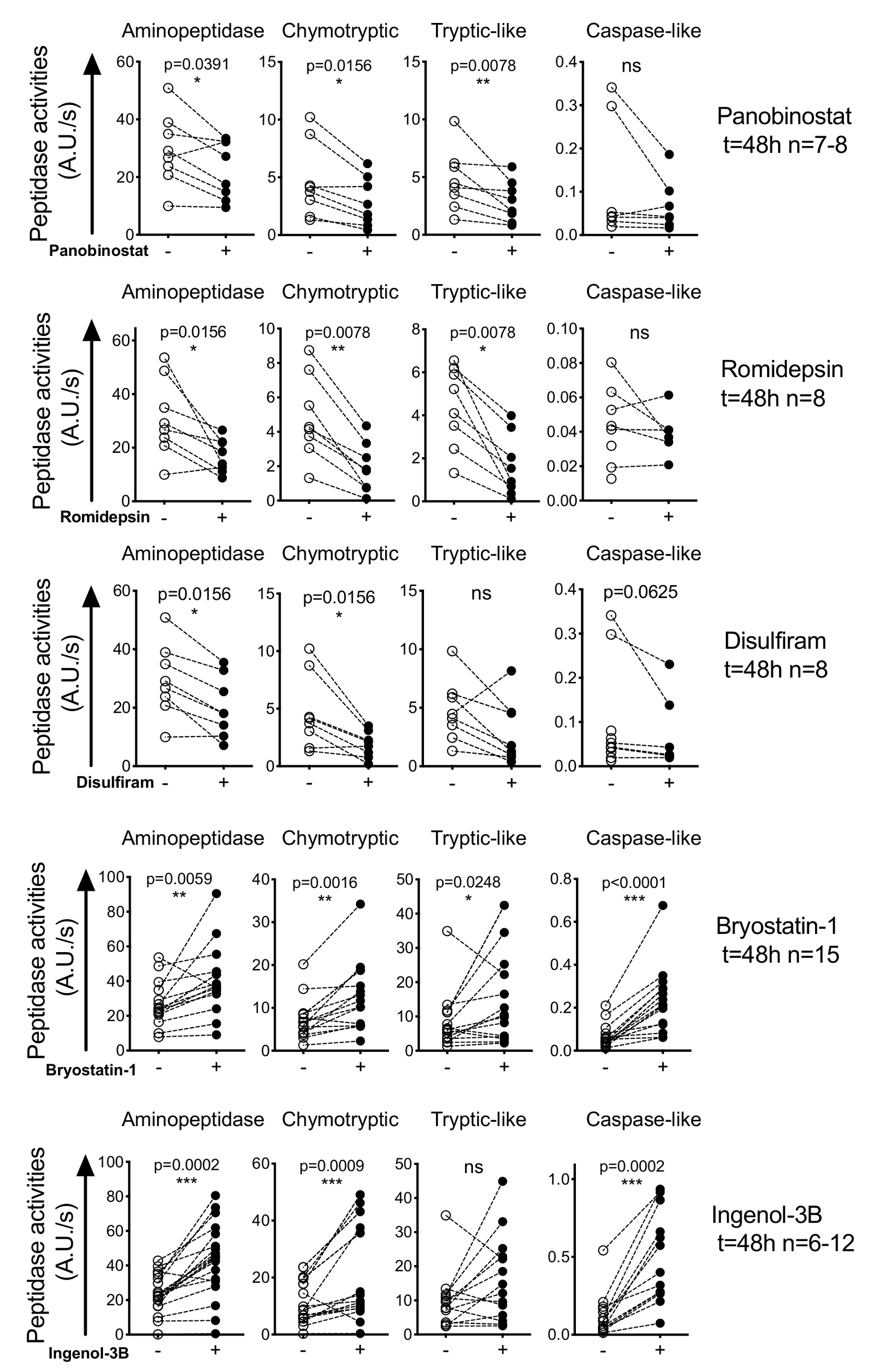

Supplement: S1 Fig — Aminopeptidase, proteasomal chymotryptic, tryptic-like and caspase-like hydrolytic activities in paired DMSO- (open circles) and LRA-treated (filled circles) primary CD4 T cells measured at 48 h post-treatment. Panobinostat, Romidepsin, Disulfiram, Bryostatin and Ingenol; results are shown for n = 7–15 healthy donors. Wilcoxon matched-pairs signed-rank t tests were performed. * p < 0.05, ** p < 0.01, *** p < 0.001, ns: not significant. (TIF) [file ppat.1008442.s001.tif]

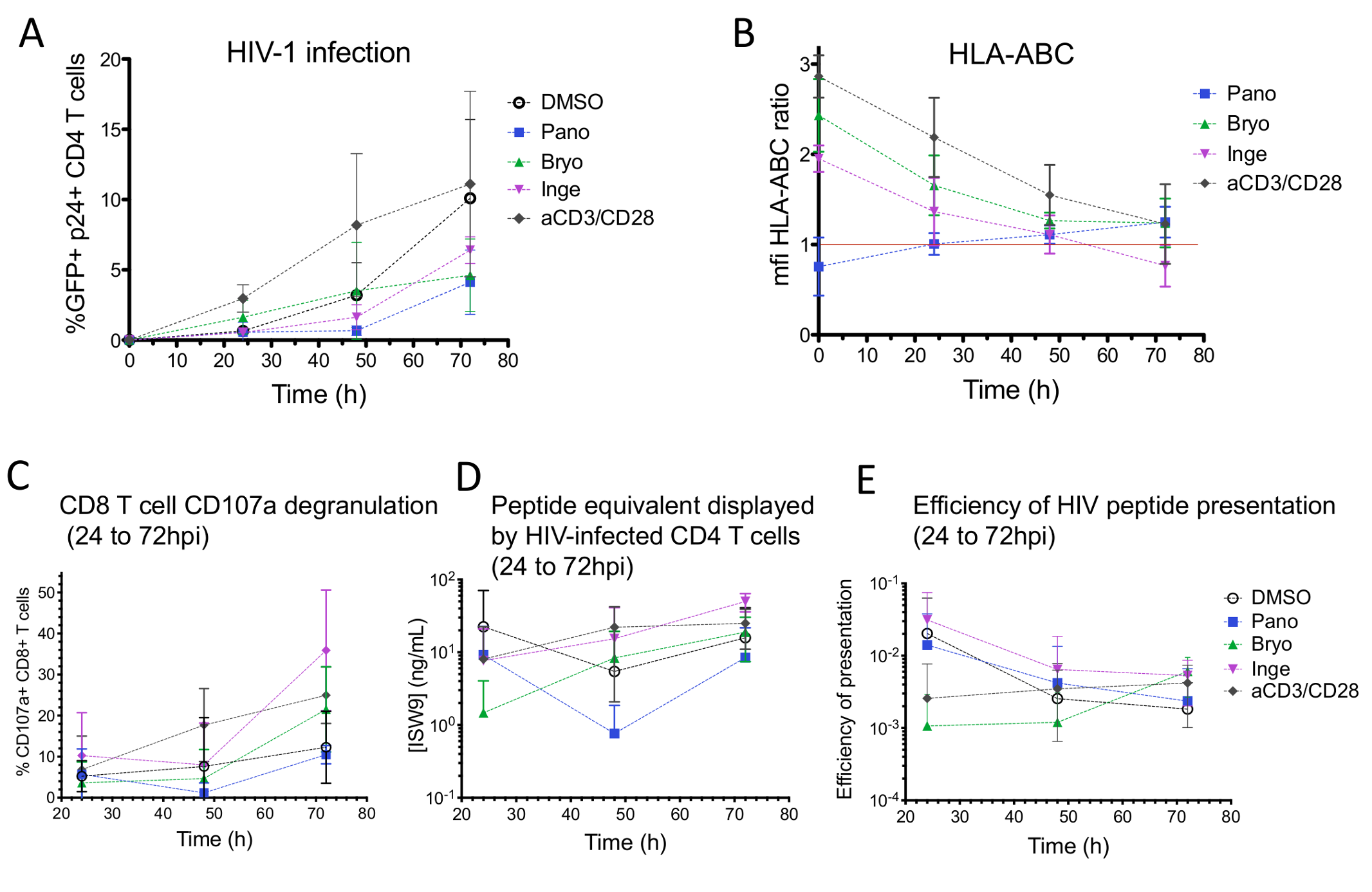

Supplement: S2 Fig — A. Primary CD4 T cells were treated with DMSO (open circles), aCD3/CD28 (grey diamonds), Panobinostat (blue squares), Bryostatin (green triangles), Ingenol (purple inverted triangles) 48 h prior to HIV-1 infection with HIV-1 NL4-3-Denv-GFP pseudotyped with VSVg. The HIV infection rate of primary CD4 T cells was assessed by flow cytometry (GFP and HIV-p24 expression) at 24, 48 and 72 h post-infection. B. HLA class I levels were assessed by flow cytometry at 0, 24, 48 and 72 h post-infection in DMSO, aCD3/CD28 and LRA-treated primary CD4 T cells. Changes upon treatment was expressed as a ratio of HLA-ABC expression of LRA- or aCD3/CD28-treated cells by their matching DMSO counterparts. Red line at ratio value = 1. C. The percentage of CD107a+ HLA-B57 ISW9-specific CD8 T after incubation with DMSO (open circles), aCD3/CD28 (grey diamonds), Panobinostat (blue squares), Bryostatin (green triangles), Ingenol (purple inverted triangles) HLA-B57+ CD4 T cells infected with HIV-1 NL4-3-ΔEnv-GFP pseudotyped with VSVg was measured at 24, 48 and 72 h post-infection at a 4:1 CTL:CD4 ratio. For each treatment (DMSO, LRA or aCD3/CD28 beads) control uninfected LRA- or aCD3/CD28 beads-treated CD4 T cells were included in the experiment and used to calculate and subtract CD107a background. D. The ISW9 peptide equivalent displayed by CD4 T cells pre-treated with DMSO, LRA or aCD3/CD28 was shown at 24, 48, 72 hpi E. The efficiency of HIV peptide presentation score at 24, 48 and 72 hpi was calculated for HLA-B57 ISW9 by dividing the relative amount of peptide presented at the cell surface by the % GFP+p24+ as indirect measurement of HIV antigen content for each treatment condition at 24, 48 and 72 h post-infection. n = 6 experiments as mean values with standard deviation. (TIF) [file ppat.1008442.s002.tif]
